# The E3 Ubiquitin Ligase COP1 Regulates Thermosensory Flowering by Triggering GI Degradation in *Arabidopsis*

**DOI:** 10.1038/srep12071

**Published:** 2015-07-10

**Authors:** Kiyoung Jang, Hong Gil Lee, Su-Jin Jung, Nam-Chon Paek, Pil Joon Seo

**Affiliations:** 1 Department of Bioactive Material Sciences and Research Center of Bioactive Materials, Chonbuk National University, Jeonju 561-756, Republic of Korea; 2 Department of Chemistry and Research Institute of Physics and Chemistry, Chonbuk National University, Jeonju 561-756, Republic of Korea; 3 Department of Plant Science, Seoul National University, Seoul 151-921, Republic of Korea

## Abstract

Floral transition is influenced by environmental factors such as light and temperature. Plants are capable of integrating photoperiod and ambient temperature signaling into their developmental program. Despite extensive investigations on individual genetic pathways, little is known about the molecular components that integrate both pathways. Here, we demonstrate that the RING finger–containing E3 ubiquitin ligase CONSTITUTIVE PHOTOMORPHOGENIC1 (COP1) acts as an integrator of photoperiod and ambient temperature signaling. In addition to the role in photoperiodic destabilization of CONSTANS (CO), COP1 also regulates temperature sensitivity by controlling the degradation of GIGANTEA (GI). *COP1*-impaired mutants showed reduced sensitivity to low ambient temperature. Notably, COP1 is more stabilized at low temperature and accelerates GI turnover in a 26S proteasome-dependent manner. The direct association of GI with the promoter of *FLOWERING LOCUS T* (*FT*) was reduced because of its ambient temperature-dependent protein stability control, and thus COP1-triggered GI turnover delays flowering at low temperatures *via* a CO-independent pathway. Taken together, our findings indicate that environmental conditions regulate the stability of COP1, and conditional specificity of its target selection stimulates proper developmental responses and ensures reproductive success.

Floral transition is a remarkable developmental transition that guarantees reproductive success. Plants utilize multiple environmental indicators including photoperiod, light intensity and quality, and temperature to determine proper timing of flowering. Temperature signals are particularly important seasonal cues. An intriguing example is vernalization, a prolonged exposure to cold, which accelerates flowering in winter-annual *Arabidopsis thaliana* ecotypes by epigenetically silencing the potent flowering repressor *FLOWERING LOCUS C* (*FLC*) so that plants synchronize their development to favorable growing seasons^1^,^2^,^3^.

Ambient temperature (non-stress temperature) also substantially affects floral transitions. In general, lower temperatures delay floral transition, whereas higher temperatures accelerate the onset of flowering^4^,^5^. This ambient temperature signaling is largely dependent on *FT*, rather than *FLC*, indicating that the temperature-dependent flowering is distinct from vernalization^4^.

Genetic contributions underlie ambient temperature responses in *Arabidopsis*, comprising the thermosensory genetic pathway^4^,^6^,^7^,^8^. The autonomous pathway components FCA and FVE are the founding members of the thermosensory pathway. Multiple alleles of *fca* and *fve* exhibit insensitive flowering phenotypes to different ambient temperatures in an *FT*-dependent manner^4^.

ACTIN-RELATED PROTEIN 6 (ARP6) substituting H2A with H2A.Z and PHYTOCHROME INTERACTING FACTOR 4 (PIF4) have also emerged as additional regulators of temperature-dependent flowering. Histone variant H2A.Z-containing nucleosomes wrap DNA more tightly, reducing accessibility of transcriptional regulatory proteins (activators and repressors) to associated target genes^9^,^10^,^11^. Notably, the histone exchange is dependent on ambient temperature. Low temperatures trigger H2A.Z incorporation, whereas H2A.Z occupancy is significantly decreased at high temperature in selective loci^12^. Consistently, *ARP6*-deficient mutants with reduced incorporation of H2A.Z show early flowering similar to plants grown at high temperatures^12^,^13^. Thus, H2A.Z depletion at high temperature appears to confer transcriptional competence and allows plants to regulate a proper temperature response by recruiting appropriate transcriptional regulators^12^,^13^. Indeed, upon high temperature-induced H2A.Z eviction, the *FT* promoter becomes more accessible for binding by the high temperature-responsive bHLH transcription factor PIF4 to stimulate floral transition^8^.

The SHORT VEGETATIVE PHASE (SVP) MADS-box transcription factor is another crucial regulator of temperature-dependent flowering^6^,^14^. Low ambient temperatures trigger SVP accumulation, which negatively regulates *FT* by binding to vCArG motifs in its promoter^6^. SVP actions are fine-tuned along with FLOWERING LOCUS M (FLM). The *FLM* gene undergoes alternative splicing, generating four splice isoforms including *FLM-*β and *FLM-*δ^15^,^16^. The relative abundance of each splice variant is determined by the ambient temperature: *FLM-β* accumulates at low temperature, whereas high temperature triggers production of *FLM-δ*^15^,^16^. The FLM-β variant forms a transcriptional repressor complex together with SVP to regulate *FT*, *SOC1*, and *TWIN SISTER OF FT* (*TSF*), suppressing floral transition at low temperature^15^. In contrast, high temperature-induced FLM-δ acts as a dominant-negative regulator and disrupts the formation of a functional complex between FLM-β and SVP^15^,^16^.

*Arabidopsis* is capable of integrating light and temperature signaling into its developmental program. Photoperiod-promotive floral transition is suppressed at low temperatures^4^. In contrast, high temperature accelerates flowering, even in short day-grown plants^17^. Furthermore, temperature-insensitive phenotypes have been observed in photoreceptor mutants^4^,^18^, suggesting that light and temperature signaling are intricately interconnected. Despite the importance of crosstalk between light and temperature signaling, the underlying molecular mechanisms remain to be elucidated.

The RING-finger E3 ligase CONSTITUTIVE PHOTOMORPHOGENIC1 (COP1) is a central regulator of light-dependent physiological processes such as photomorphogenesis, circadian oscillation, and floral transition^19^,^20^,^21^. In this work, we report that COP1 is also involved in ambient temperature-dependent flowering. COP1 is stabilized at low ambient temperatures and promotes degradation of GI that directly regulates *FT* expression. Consistently, *COP1*-deficient mutants exhibited reduced sensitivity to changes in ambient temperatures in an *FT*-dependent manner. Our findings provide biological insights into how temperature and photoperiod signals are integrated.

## Results

### *cop1* mutants are insensitive to changes in ambient temperature in flowering

**Figure 1:**
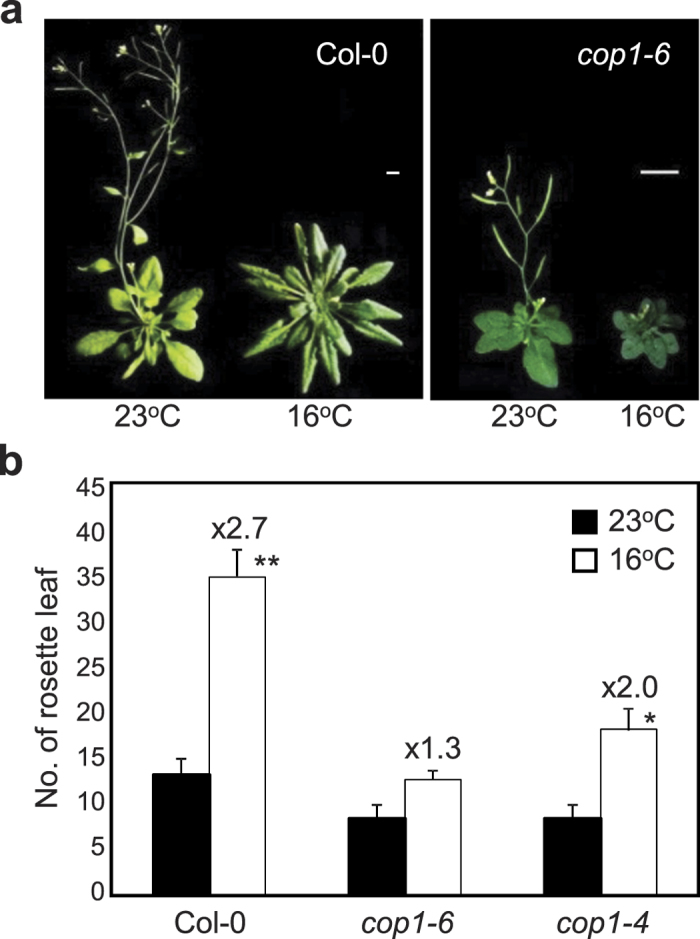
*cop1–6* is insensitive to low ambient temperature. (**a**) Flowering phenotypes of *cop1–6*. Plants were grown for 7 weeks at 23 ^°^C or at 16 ^°^C under long-day conditions (LDs). Scale bar = 1 cm. (**b**) Flowering times of *cop1–6* and *cop1–4* at 23 ^°^C and 16 ^°^C. Flowering time was measured by counting the total number of rosette leaves at flowering initiation. Three biological replicates were averaged and statistically significant differences between the values of each genotype at 23 ^°^C and 16 ^°^C are indicated by asterisks (Student’s *t*-test, **P < 0.01; *P < 0.05). Bars indicate standard error of the mean. The numbers above the bars indicate the ratio of flowering at 16 ^°^C and 23 ^°^C (16 ^°^C/23 ^°^C).

Ambient temperature signaling is closely intertwined with photoperiod signaling^7^,^17^,^22^,^23^. In a preliminary attempt to investigate light and temperature signaling crosstalk, we assessed light signaling mutants for their sensitivity to low ambient temperature. Of the light-signaling mutants examined, the *COP1*-impaired *cop1–6* mutant showed reduced sensitivity to changes in ambient temperature under long day (LD) conditions. Flowering of wild-type (Col-0) plants was substantially delayed at 16 ^°^C with a flowering time ratio (16 ^°^C/23 ^°^C) of approximately 2.7 (Fig. 1a,b), which was consistent with previous observations^4^. However, *cop1–6* mutant exhibited early flowering independent of ambient temperatures, with a flowering time ratio of approximately 1.3 (Fig. 1a,b).

To determine the role of COP1 in thermosensory flowering, we obtained additional *cop1-4* mutant and measured flowering time under different temperature conditions^21^. *cop1–4* mutant exhibited a significant decrease in the sensitivity to low ambient temperature similar to *cop1–6* mutant (Fig. 1b), suggesting that COP1 is involved in ambient temperature-dependent floral transition. The slight differences of *cop1–4* and *cop1–6* in temperature sensitivity might be due to allelic variations^20^. Because of its strong phenotypes, we employed *cop1–6* mutant for subsequent analyses.

### *FT* is required for COP1 action

Ambient temperature signals are integrated into the flowering integrator *FT*, and thus we next examined whether COP1 affects *FT* expression to achieve proper developmental responses to ambient temperatures.

**Figure 2:**
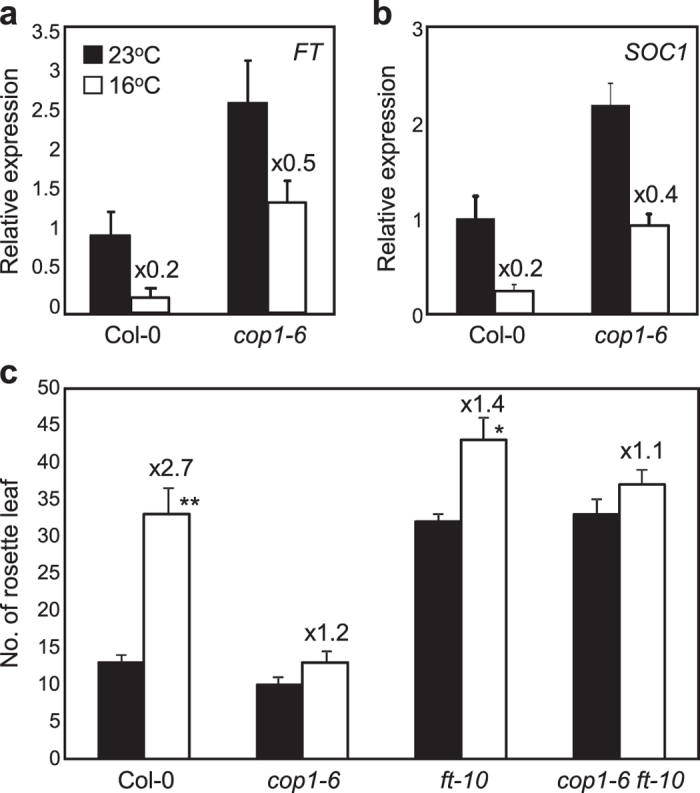
*FT* is required for thermal insensitivity of *cop1-6*. Relative expression of *FT* (**a**) and *SOC1* (**b**) at zeitgeber time (ZT) 16 was analyzed by RT-qPCR. Twelve-day-old seedlings grown under LDs were used to analyze transcript accumulation. Bars indicate standard error of the mean. The numbers above the bars indicate the ratio of expression at 16 ^°^C and 23 ^°^C (16 ^°^C/23 ^°^C). (**c**) Flowering times of *cop1-6*, *ft-10* and *cop1–6 ft-10* at 23 ^°^C and 16 ^°^C. Flowering time was measured by counting the total number of rosette leaves at flowering initiation. Three biological replicates were averaged and statistically significant differences between the values of each genotype at 23 ^°^C and 16 ^°^C are indicated by asterisks (Student’s *t*-test, **P < 0.01; *P < 0.05). Bars indicate standard error of the mean. The numbers above the bars indicate the ratio of expression at 16 ^°^C and 23 ^°^C (16 ^°^C/23 ^°^C).

*FT* expression was substantially suppressed at low temperatures in wild-type plants with a ratio (16 ^°^C/23 ^°^C) of 0.2, whereas the temperature-dependent changes in *FT* expression were significantly reduced in *cop1–6* mutant (Fig. 2a). *SOC1* is another flowering integrator that acts partially downstream of *FT* 24. To examine whether the altered expression of *FT* was relevant to a flowering pathway, we analyzed expression of *SOC1*. Similar to *FT* expression, transcript accumulation of *SOC1* was also reduced in wild-type plants at low ambient temperature, but the change was significantly reduced in *cop1–6* mutant (Fig. 2b).

To further evaluate the genetic hierarchy between *FT* and *COP1*, we crossed *ft-10* with *cop1–6*. Although a stunted growth phenotypes were observed (Fig. S1), the flowering phenotype of the *cop1–6 ft-10* double mutant was comparable to that of *ft-10* regardless of ambient temperatures (Fig. 2c), indicating that *FT* acts downstream of *COP1* in a thermosensory pathway.

### COP1 stability is controlled by ambient temperature

We next asked how COP1 perceives ambient temperature signals. To that end, we measured transcript accumulation of *COP1* in seedlings grown at different ambient temperatures. Low temperature resulted in reduced transcript accumulation of *COP1*, whereas expression of *COP1* was unchanged at high temperatures (Fig. S2). Since reduced expression of *COP1* cannot explain the early flowering phenotype of *cop1–6* mutant at low temperatures, it can be considered that *COP1* expression might be associated with feedback regulation of protein accumulation. It is likely that at low ambient temperature, *COP1* transcription is reduced to maintain homeostasis for increased COP1 levels.

**Figure 3:**
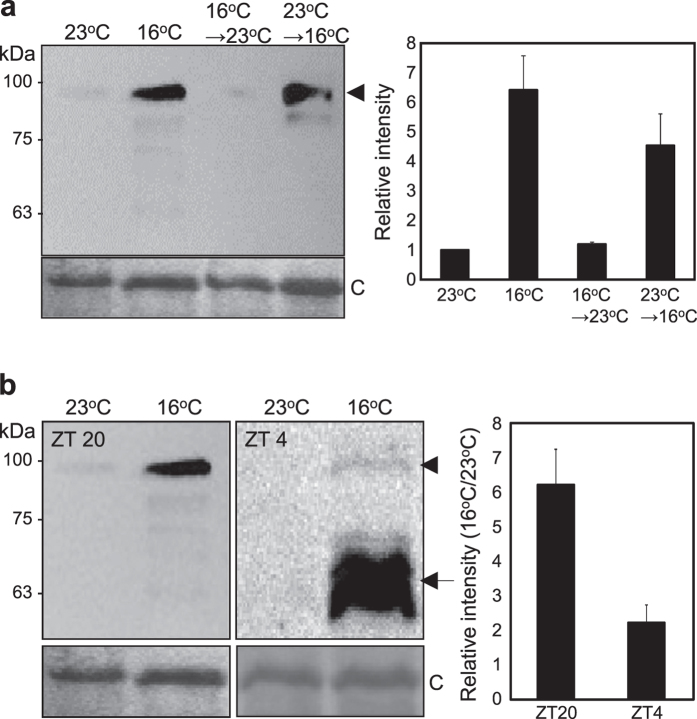
COP1 stability is controlled by ambient temperature. (**a**) COP1 accumulation at different ambient temperatures. Twelve-day-old 35S:*COP1-TAP* seedlings grown at 23 ^°^C or 16 ^°^C were collected at the end of night (ZT24) for immunoblot analysis. To minimize secondary effects caused by growth rate difference, 10-day-old seedlings grown at 16 ^°^C or 23 ^°^C under LDs were transferred to different ambient temperatures and incubated for 2 days. (**b**) Accumulation of COP1 at 16 ^°^C in light and dark. Twelve-day-old seedlings grown under LDs were harvested at ZT20 or ZT4. COP1-TAP proteins (arrowheads) were detected immunologically using an anti-MYC antibody. Low molecular bands (arrows) might be degradation products. A part of Coomassie blue–stained gel (**C**) is shown as a loading control. Bands from three blots were quantified using Image J software and averaged (right panels).

To test this hypothesis, we employed the 35S:*COP1-TAP* transgenic plants^21^ grown at different ambient temperatures. Notably, COP1 protein levels increased significantly at low temperature compared with its levels at 23 ^°^C (Fig. 3a). The temperature effect on COP1 accumulation was observed in both light and dark periods of the day, while it was more prominent in the dark (Fig. 3b). COP1 stability seems to be controlled by multiple environmental factors including light and temperature, and the distinct degradation patterns of COP1 might be owing to the different destructive mechanisms depending on the surrounding environments.

To minimize the secondary effect caused by reduced growth rates at low temperature, we transferred 23 ^°^C-grown plants to 16 ^°^C and *vice versa* and incubated them for 2 days (Fig. 3a). Short-term exposure to low temperature also stimulated accumulation of COP1. In contrast, COP1 degradation was unchanged at high temperature (Fig. S3), indicating that the stability of COP1 is primarily regulated by low ambient temperature.

### Floral transition at low ambient temperature is regulated in part by COP1-GI module

CO is a representative proteolytic target of COP1, and thus we tested if CO activity is regulated by ambient temperatures. However, transcript and protein accumulation of CO were largely independent of changes in ambient temperature (Fig. S4 and S5). In addition, it has been previously demonstrated that misexpression of *CO* do not influence temperature sensitivity in floral transition^4^,^25^, suggesting that CO is not a component of the thermosensory pathway.

**Figure 4:**
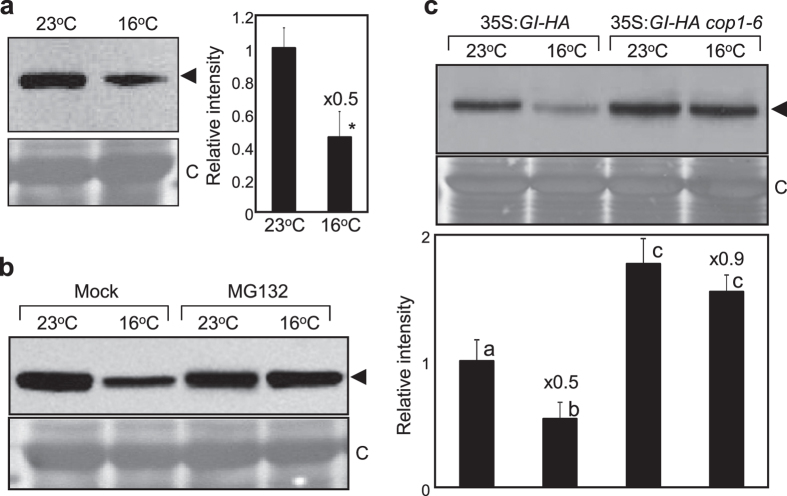
COP1 triggers GI turnover. Twelve-day-old 35S:*GI-HA* seedlings grown at 23 ^°^C or 16 ^°^C under LDs were collected at ZT24. GI-HA proteins (arrowheads) were detected immunologically using an anti-HA antibody. A part of the Coomassie blue–stained gel (**C**) is shown as a loading control. (**a**) GI accumulation at 23 ^°^C and 16 ^°^C. (**b**) Effects of MG132 on GI stability. (**c**) Low temperature-triggered GI turnover in *cop1-6*. Bands from three blots were quantified using Image J software and averaged. Statistical significance of the measurements was determined by a Student’s *t*-test (*P < 0.05) or one-way anova with Fisher’s *post hoc* test (*P *< 0.05). The numbers above the bars indicate the ratio of expression at 16 ^°^C and 23 ^°^C (16 ^°^C/23 ^°^C).

COP1 is also involved in the regulation of GI stability along with ELF3^21^. We therefore investigated whether COP1 regulates protein turnover of GI at low temperatures. To this end, we employed the 35S:*GI-HA* transgenic plants^26^ grown at different ambient temperatures to monitor GI accumulation. The stability of GI protein was markedly reduced at 16 ^°^C compared with that at 23 ^°^C (Fig. 4a), and the reduced protein accumulation was recovered by treatment with MG132, a potent inhibitor of the 26S proteasome (Fig. 4b), suggesting that an ubiquitination process underlies temperature-dependent GI accumulation. To further support a role of COP1 in GI turnover at low temperature, the 35S:*GI-HA* transgenic plants were crossed with *cop1–6* mutant. In the 35S:*GI-HA cop1–6* plants, GI accumulation was elevated compared with that of 35S:*GI-HA* plants and was not sensitive to changes in ambient temperature (Fig. 4c), indicating that COP1 is an unequivocal regulator of GI turnover at low temperature.

The ELF3 adaptor protein is also proteolytically degraded by COP1 in order to control GI turnover^21^. However, proteolysis of ELF3 was not increased at low temperatures (Fig. S6), although it appeared that ELF3 is required for the proper response to changes in ambient temperature (Fig. S7 and S8)^7^. Different molecular mechanisms likely underlie regulation of ELF3 activity at different ambient temperatures (see Discussion).

**Figure 5:**
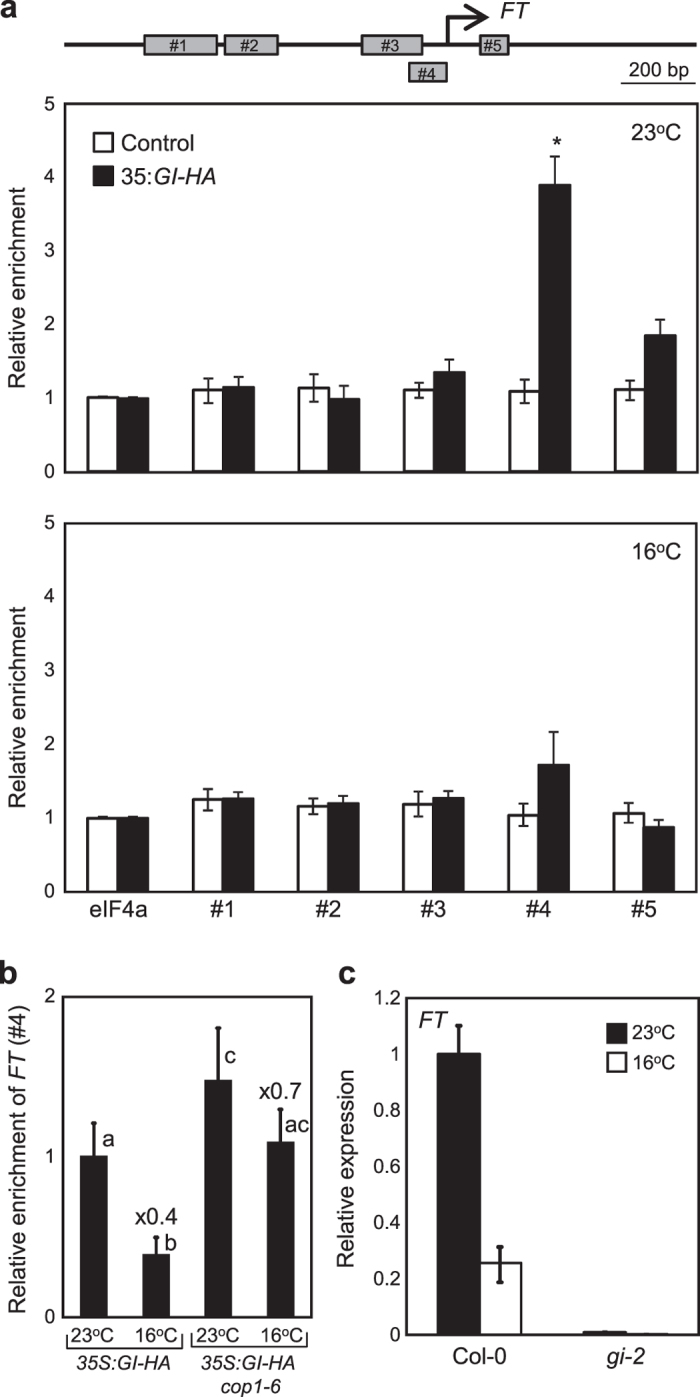
Association of GI to the *FT* promoter is dependent on ambient temperature. (**a**) ChIP assays. Five qPCR amplicons on the *FT* promoter are shown. Twelve-day-old 35S:*GI-HA* plants grown at 23 ^°^C or 16 ^°^C under LDs were harvested at ZT24 for ChIP analysis. Statistical significance of the measurements was determined by a Student’s *t*-test (*P < 0.05). (**b**) ChIP analysis of GI binding to *FT* in *cop1-6* at ZT24. Different letters represent a significant difference at *P *< 0.05 (one-way anova with Fisher’s *post hoc* test). The numbers above the bars indicate the ratio of expression at 16 ^°^C and 23 ^°^C in each genotype (16 ^°^C/23 ^°^C). (**c**) Relative expression of *FT* in *gi-2* at 23 ^°^C and 16 ^°^C. Twelve-day-old seedlings grown under LDs were harvested at ZT16 to analyze transcript accumulation. Bars indicate standard error of the mean. The numbers above the bars indicate the ratio of expression at 16 ^°^C and 23 ^°^C (16 ^°^C/23 ^°^C).

GI binds directly to the *FT* promoter^27^. Thus, we investigated whether the binding of GI to the *FT* promoter is regulated by ambient temperature. The 35S:*GI-HA* transgenic plants were grown at 23 ^°^C and 16 ^°^C. Total protein extracts from the control and 35S:*GI-HA* transgenic plants were immuno-precipitated with an anti-HA antibody. DNA bound to epitope-tagged GI proteins was analyzed by quantitative PCR (qPCR) assays. The ChIP analysis showed that the *FT* promoter was enriched by GI (Fig. 5a) as previously reported^27^. However, the GI association with the *FT* promoter was substantially reduced at low temperature, possibly due to reduced stability of GI protein (Fig. 5a). The reduced GI binding to the *FT* promoter at low temperature was impaired in *cop1–6* mutant (Fig. 5b). To further convince this observation, we analyzed *FT* expression in *gi-2* mutant at low ambient temperature. While *FT* expression was substantially reduced at 16 ^°^C in wild-type background, constitutively reduced *FT* expression was observed in *gi-2* mutant regardless of ambient temperatures (Fig. 5c), indicating the significant role of COP1-GI module in thermosensory flowering.

**Figure 6:**
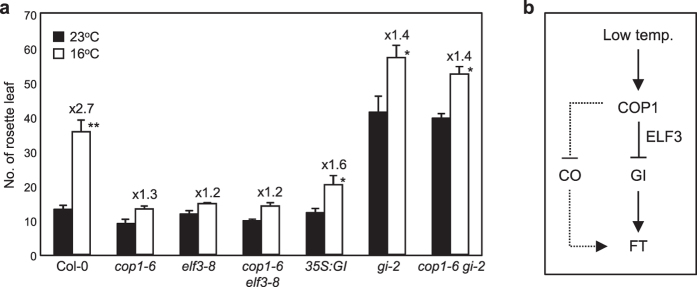
*GI* is epistatic to *COP1* in thermosensory flowering. (**a**) Flowering times of mutants and transgenic plants. Flowering time was measured by counting the total number of rosette leaves at flowering initiation. Three biological replicates were averaged and statistically significant differences between the values at 23 ^°^C and 16 ^°^C are indicated by asterisks (Student’s *t*-test, **P < 0.01; *P < 0.05). Bars indicate standard error of the mean. The numbers above the bars indicate the ratio of flowering at 16 ^°^C and 23 ^°^C (16 ^°^C/23 ^°^C). (**b**) Working diagram of COP1-mediated thermosensory flowering. Low temperature-triggered COP1 accumulation induces GI degradation. Thus, GI binding to the *FT* promoter is reduced and floral transition is delayed in a CO-independent manner.

To confirm the genetic relationship between *GI* and *COP1*, we crossed between *gi-2* and *cop1–6* mutants. Under normal growth conditions, the flowering time of *gi-2 cop1–6* mutant was comparable to that of *gi-2* mutant (Fig. 6a). Furthermore, the temperature responsiveness of *gi-2 cop1–6* and *gi-2* mutants was also comparable, indicating that they act on the same genetic pathway.

Taken together, COP1 is a central regulator that integrates photoperiod and temperature signaling. In addition to its photoperiodic regulation of CO, COP1 also regulates temperature-dependent GI stability (Fig. 6b). At low temperature, COP1 is stabilized and triggers GI degradation even in inductive LD conditions. COP1 stability regulated by both photoperiod and ambient temperature facilitates to make integrative decisions for determining timing to flower, and the conditional specificity of COP1 to its targets ensures proper developmental transitions in plants.

## Discussion

### Controlled protein turnover and temperature signaling

Covalent attachment of ubiquitin proteins is a common means of modulating protein stability. The ubiquitination process is mediated by the sequential action of three enzymes: ubiquitin-activating enzymes (E1), ubiquitin-conjugating enzymes (E2), and ubiquitin ligases (E3). The *Arabidopsis* genome encodes 2 E1, >37 E2, and >1,500 E3 enzymes^28^,^29^. Consistent with the large number of enzymes in the ubiquitination process, they are involved in diverse aspects of plant growth and development including cell cycle progression, hormone responses, and plant responses to biotic and abiotic stresses^30^,^31^,^32^.

Although no E3 ligases involved in thermosensory flowering have been identified to date, controlled protein turnover is likely crucial for temperature-dependent floral transition. For example, protein turnover of SVP is accelerated at high temperature, and SVP degradation is dependent on the 26S proteasome activity^15^.

In this study, we report that COP1 is implicated in ambient temperature-dependent floral transition. COP1 is more stabilized at low temperature and subsequently accelerates protein turnover of GI, which in turn down-regulates *FT* expression to determine proper timing to flower^27^. Consistently, *cop1* and *gi* mutants are almost insensitive to changes in ambient temperatures.

It seems likely that GI and SVP antagonistically regulate *FT* expression by binding to the *FT* promoter depending on ambient temperatures. At low temperatures, SVP protein is stabilized, while GI is degraded by a COP1-mediated ubiquitination process. At high temperatures, a reverse pattern would be relevant for early flowering, whereby GI would be stabilized with reduced SVP activity. Collectively, balanced transcriptional regulation of *FT* is achieved by coordinated control of the transcriptional activator GI and repressor SVP, and temperature-dependent protein turnover is a key molecular mechanism underlying reestablishment of balanced accumulation of these proteins.

### Integration of photoperiod and temperature information

Ambient temperature and photoperiod pathways are closely intertwined. Late flowering in short days can be overcome by growing at high temperature^17^. In contrast, long day-triggered early flowering is compromised at low temperature^4^. Furthermore, these two genetic pathways are largely dependent on the flowering pathway integrator *FT*^7^,^23^, suggesting the presence of intensive crosstalk.

Our results suggest that regulation of COP1 stability is at the center of this crosstalk. COP1 is a well-known E3 ligase that participates in photoperiod flowering, which is particularly stabilized during night and rapidly degrades accumulated CO^19^. This study provides evidence that COP1 is also involved in thermosensory flowering. At low temperature, COP1 is more stabilized and down-regulates GI stability. Since GI directly binds to the *FT* promoter^27^, the GI abundance is closely associated with transcript accumulation of *FT* in a CO-independent manner. Notably, increased stability of COP1 was also relevant with light period, thereby serving to integrate photoperiod and ambient temperature signaling.

At low temperatures, protein stability of ELF3 is largely unaffected despite the enhanced activity of COP1 (Fig. S6). It seems contrary to the previous observation that COP1 induces proteolysis of ELF3 concomitant with GI degradation^21^. However, these results may suggest that ELF3 is either necessary but not sufficient for COP1-mediated ubiquitination of GI, or that ELF3 is not a rate-limiting factor for the activity of COP1.

It is known that CO is not associated with thermosensory flowering^4^,^25^. Consistently, we also showed that protein turnover of CO is not responsive to changes in ambient temperatures. However, it is currently unclear how low temperature-activated COP1 specifically triggers GI turnover. Additional molecular components (i.e. ELF3) that guide target selection depending on environmental conditions are likely involved, although further studies are required to develop a comprehensive understanding of the signaling network underlying the environmental regulation of flowering.

Collectively, we show that COP1 perceives light and temperature signals at the level of protein accumulation. At normal growth temperature, COP1 primarily induces CO degradation during night-time. At low temperature, COP1 is substantially stabilized under both light and dark conditions, triggering GI turnover without affecting CO stability. These results demonstrate that COP1 acts as a central player in the integration of ambient temperature and photoperiod signaling and ensures proper floral transition to minimize reproductive failure.

## Methods

### Plant materials and growth conditions

*Arabidopsis thaliana* (Columbia-0 ecotype) was used for all experiments, unless specified otherwise. Plants were grown under long day conditions (LDs; 16-h light/8-h dark cycles) with cool white fluorescent light (100 μmol photons m^-2^ s^-1^) at 23 ^°^C and 16 ^°^C. The *cop1-4*, *cop1-6*, *elf3-8*, *ft-10*, and *gi-2* mutants were previously reported^6^,^21^,^33^,^34^. The transgenic plants 35S:*COP1-TAP* and 35S:*GI-HA* were used as described previously^21^,^26^.

### Flowering time measurements

Plants were grown on soil at either 23 ^°^C or 16 ^°^C under LDs. Flowering time was measured by counting the total number of rosette leaves at flowering initiation. A total of 40 to 50 plants were measured and averaged for each plant group. Statistical significance was determined using Student’s *t*-test (**P < 0.01; *P < 0.05).

### Quantitative real-time RT-PCR analysis

Total RNA was extracted using TRI agent (Takara Bio, Singa, Japan) according to the manufacturer’s recommendations. Reverse transcription (RT) was performed using Moloney Murine Leukemia Virus (M-MLV) reverse transcriptase (Dr. Protein, Seoul, South Korea) with oligo(dT18) to synthesize first-strand cDNA from 2 μg of total RNA. Total RNA samples were pretreated with an RNAse-free DNAse. cDNAs were diluted to 100 μL with TE buffer, and 1 μL of diluted cDNA was used for PCR amplification.

Quantitative RT-PCR reactions were performed in 96-well blocks using the Step-One Plus Real-Time PCR System (Applied Biosystems). The PCR primers used are listed in Table S1. The values for each set of primers were normalized relative to the *EUKARYOTIC TRANSLATION INITIATION FACTOR 4A1* (*eIF4A*) gene (At3g13920). All RT-qPCR reactions were performed in three technical replicates using total RNA samples extracted from three independent biological replicate samples. The comparative ΔΔC_T_ method was employed to evaluate relative quantities of each amplified product in the samples. The threshold cycle (C_T_) was automatically determined for each reaction by the system set with default parameters. The specificity of the RT-qPCR reactions was determined by melt curve analysis of the amplified products. A melting curve was constructed by measuring the fluorescence continuously when heating from 60 to 95 ^°^C at the rate of 0.5 ^°^C/sec.

### Immunoblot analysis

Harvested plant materials were ground in liquid nitrogen, and total cellular extracts were suspended in SDS-PAGE sample loading buffer. The protein samples were then analyzed by SDS/PAGE (10% gels) and blotted on to Hybond-P+ membranes (Amersham-Pharmacia). The epitope-tagged proteins were immunologically detected using anti-MYC or anti-HA antibodies (Millipore, Billerica, MA, USA).

### Chromatin immunoprecipitation (ChIP)

ChIP assays were performed as previously described^35^. Briefly, epitope-tagged transgenic plants, anti-MYC and anti-HA antibodies (Millipore) and salmon sperm DNA/protein A agarose beads (Millipore) were used for chromatin immunoprecipitation. DNA was purified using phenol/chloroform/isoamyl alcohol and sodium acetate (pH 5.2). The level of precipitated DNA fragments was quantified by quantitative real-time PCR using specific primer sets (Table S2). Values were normalized according to input DNA level. Values for control plants were set to 1 after normalization against *eIF4a* for quantitative PCR analysis.

## Additional Information

**How to cite this article**: Jang, K. *et al.* The E3 Ubiquitin Ligase COP1 Regulates Thermosensory Flowering by Triggering GI Degradation in *Arabidopsis*. *Sci. Rep.* **5**, 12071; doi: 10.1038/srep12071 (2015).

## Supplementary Material

Supplementary Information
