# Supplementary material for: The E3 Ubiquitin Ligase COP1 Regulates Thermosensory Flowering by Triggering GI Degradation in Arabidopsis
Source: Sci Rep. 2015 Jul 10;5:12071. doi: 10.1038/srep12071 (PMC4498190; doi:10.1038/srep12071)
Supplement: Supplementary Information [file srep12071-s1.pdf]

SUPPLEMENTARY INFORMATION

The E3 Ubiquitin Ligase COP1 Regulates Thermosensory Flowering by Triggering GI Degradation in *Arabidopsis*

Kiyoung Jang, Hong Gil Lee, Su-Jin Jung, Nam-Chon Paek & Pil Joon Seo

Supplementary Figures

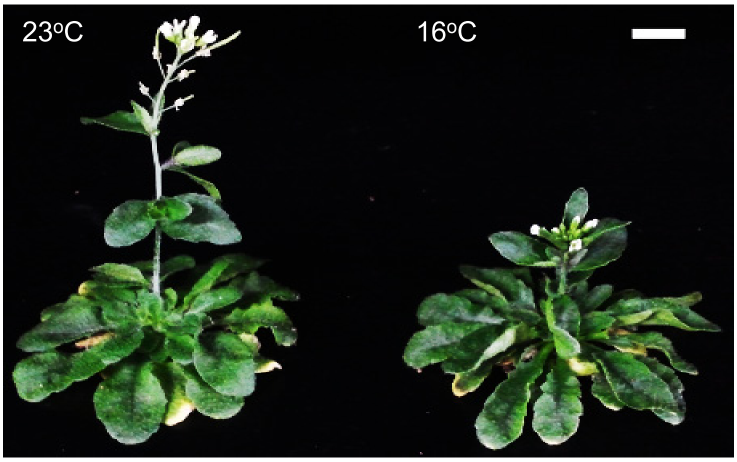

Supplementary Figure S1. Flowering phenotypes of *cop1-6 ft-10*.
Eleven-week-old *cop1-6 ft-10* plants grown at 23°C and 16°C under long day conditions (LDs, 16-h light/8-h dark) were photographed. Scale bar = 1 cm.

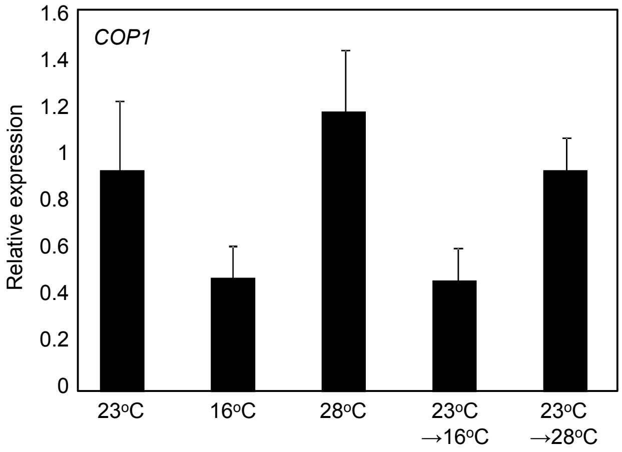

Supplementary Figure S2. Expression of *COP1* at different ambient temperatures.
Twelve-day-old wild-type seedlings grown at different ambient temperatures under LDs were used to analyze transcript accumulation of *COP1*. To minimize secondary effects caused by different growth rates, 10-day-old seedlings grown at 23°C under LDs were transferred to different ambient temperatures and incubated for 2 days. Transcript accumulation was analyzed by quantitative real-time RT-PCR (RT-qPCR). Three biological replicates were averaged. Bars indicate standard error of the mean.

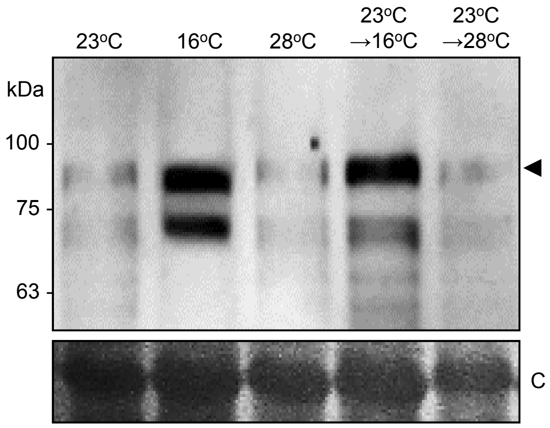

Supplementary Figure S3. COP1 accumulation at different ambient temperatures.
Twelve-day-old 35S:*COP1-TAP* seedlings grown at different ambient temperatures under LDs were used for immunoblot analysis. To minimize secondary effects caused by different growth rates, 10-day-old seedlings grown at 23°C under LDs were transferred to different ambient temperatures and incubated for 2 days. The COP1-TAP proteins (arrowheads) were detected immunologically using an anti-MYC antibody. A part of the Coomassie blue–stained gel (C) is shown as a loading control.

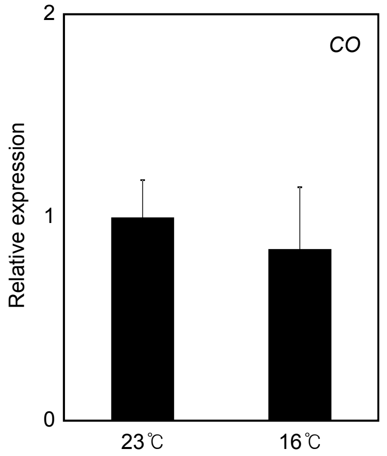

Supplementary Figure S4. Relative expression of *CO* in wild-type plants grown at 23ºC and 16ºC. Twelve-day-old seedlings grown at 23ºC and 16ºC under LDs were used to analyze transcript accumulation of *CO*. Transcript accumulation was analyzed by RT-qPCR. Three biological replicates were averaged. Bars indicate standard error of the mean.

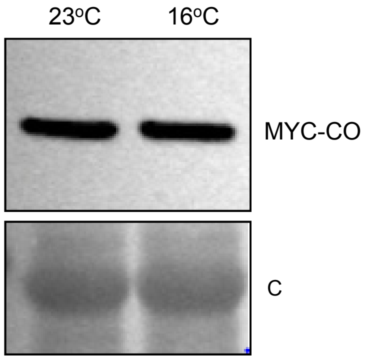

Supplementary Figure S5. Accumulation of CO at 23°C and 16°C.
Twelve-day-old 35S:*MYC-CO* seedlings grown at 23°C or 16°C under LDs were used for immunoblot analysis. The CO proteins were detected immunologically using an anti-MYC antibody. A part of the Coomassie blue–stained gel (C) is shown as a loading control.

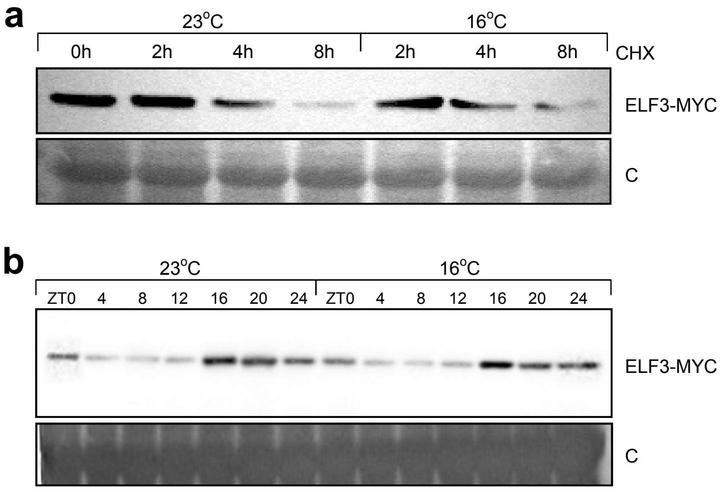

Supplementary Figure S6. ELF3 accumulation at 23°C and 16°C.

ELF3 proteins were detected immunologically using an anti-MYC antibody. A part of the Coomassie blue-stained gel (C) is shown as a loading control.

(**a**) Cycloheximide treatment. Twelve-day-old 35S:*ELF3-MYC* seedlings grown at 23°C under LDs were transferred to MS-liquid medium supplemented with 50 μM cycloheximide (CHX) and incubated at 23°C and 16°C for the indicated time period. h, hours.

(**b**) Diurnal accumulation of ELF3 at 23°C and 16°C. Twelve-day-old 35S:*ELF3-MYC* seedlings grown at 23°C and 16°C under LDs were used for immunoblot analysis. ZT, zeitgeber.

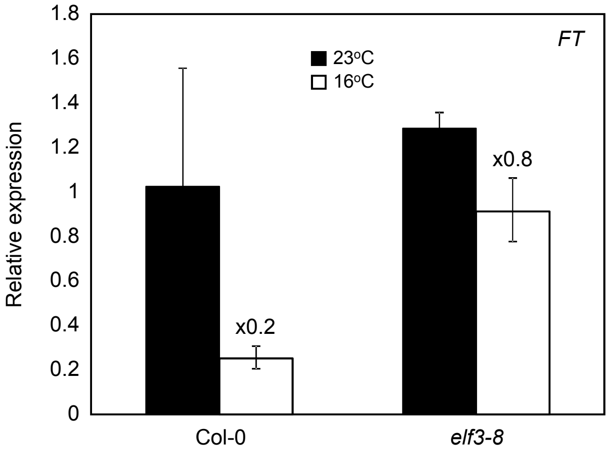

Supplementary Figure S7. Relative expression of *FT* in wild-type and *elf3-8* mutant seedlings grown at 23°C and 16°C.
Twelve-day-old seedlings grown at 23°C and 16°C under LDs were used to analyze transcript accumulation of *FT*. Transcript accumulation was analyzed by RT-qPCR. Three biological replicates were averaged. Bars indicate standard error of the mean. The numbers above the bars indicate the ratio of expression at 16°C and 23°C (16°C/23°C).

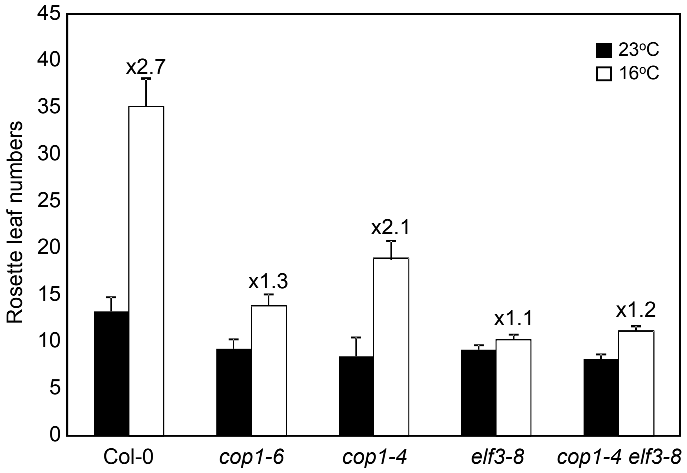

Supplementary Figure S8. Flowering phenotypes of *cop1* and *elf3* mutants at 23°C and 16°C. Flowering time was measured by counting total number of rosette leaves at flowering initiation. Three biological replicates were averaged. Bars indicate standard error of the mean. The numbers above the bars indicate the ratio of rosette leaf numbers at 16°C and 23°C (16°C/23°C).

Supplementary Table

Primer	Usage	Sequence
eIF4a-F	RT-qPCR	5′-TGACCACACAGTCTCTGCAA
eIF4a-R	RT-qPCR	5′-ACCAGGGAGACTTGTTGGAC
FT-F	RT-qPCR	5′-GGTGGAGAAGACCTCAGGAA
FT-R	RT-qPCR	5′-GGTTGCTAGGACTTGGAACATC
SOC1-F	RT-qPCR	5′-TGAGGGGCAAAACTCAGATG
SOC1-R	RT-qPCR	5′-TCTTGCATATTGGAGCTGGC
COP1-F	RT-qPCR	5′-GAGGCAGGAAGCAAGTGTGA
COP1-R	RT-qPCR	5′-CGACCGCAATGTAGTTGCTT

Supplementary Table S1. Primers used in this study.
RT-qPCR primers were designed using the Primer Express Software installed into the Applied Biosystems 7500 Real-Time PCR System. The sizes of PCR products ranged from 80 to 300 nucleotides in length. F, forward primer; R, reverse primer.

Primer	Sequence
FT(#1)-F	5′-TCTTATAGTACTGAGCTCTCCTGTCCA
FT(#1)-R	5′-CCATAGCCTAACAACTGTAGGAA
FT(#2)-F	5′-TTCATCTTTGAACTTAAGAAATGCTC
FT(#2)-R	5′-TTTTTATAAACAAGCGGCCATA
FT(#3)-F	5′-TTGGCGGTACCCTACTTTTT
FT(#3)-R	5′-TTTCGGATTTGCATTAACTCG
FT(#4)-F	5′-AAGACGACAATGTGTGATGTACG
FT(#4)-R	5′-TGATCTTGAACAAACAGGTGGT
FT(#5)-F	5′-GGTGGAGAAGACCTCAGGAA
FT(#5)-R	5′-GTGGGGCATTTTTAACCAAG

Supplementary Table S2. Primers used in chromatin immunoprecipitation (ChIP) assays.
F, forward primer; R, reverse primer.
